# Phosphorylation of Ser^78 ^of Hsp27 correlated with HER-2/*neu *status and lymph node positivity in breast cancer

**DOI:** 10.1186/1476-4598-6-52

**Published:** 2007-08-14

**Authors:** Daohai Zhang, Lee Lee Wong, Evelyn SC Koay

**Affiliations:** 1 Department of Laboratory Medicine, National University Hospital, 5 Lower Kent Ridge Road, 119074, Singapore; 2 Department of Pathology, Yong Loo Lin School of Medicine, National University of Singapore, 119074, Singapore

## Abstract

**Background:**

Abnormal amplification/expression of HER-2/*neu *oncogene has been causally linked with tumorigenesis and metastasis in breast cancer and associated with shortened overall survival of patients. Recently, heat shock protein 27 (Hsp27) was reported to be highly expressed in HER-2/*neu *positive tumors and cell lines. However, putative functional links between phosphorylation of Hsp27 with HER-2/*neu *status and other clinicopathological features remain to be elucidated.

**Results:**

Comparative phosphoproteomic studies of HER-2/*neu *positive and -negative breast tumors revealed that Hsp27, one of the identified phosphoproteins, was highly phosphorylated in HER-2/*neu *positive tumors. The extent of Hsp27 phosphorylation at its Ser^15^, Ser^78 ^and Ser^82 ^residues were further evaluated with site-specific antibodies in tumor samples by tissue lysate array- and tissue microarray-based analyses, and in the BT474 breast cancer cell line treated with heregulin α1 (HRG α1) or the p38 MAPK inhibitor, SB203580. The tissue lysate array study indicated that only the level of pSer^78 ^in HER-2/*neu *positive tumors was more than 2-fold that in HER-2/*neu *negative tumors. Treatment of BT474 cells with HRG α1 and SB203580 indicated that Ser^78 ^phosphorylation was mainly regulated by the HER-2/*neu*-p38 MAPK pathway. Immunohistochemical staining of sections from a tissue microarray with 97 breast tumors showed that positive staining of pSer^78 ^significantly correlated with HER-2/*neu *(*p *= 0.004) and lymph node positivity (*p *= 0.026).

**Conclusion:**

This investigation demonstrated the significant correlation of enhanced phosphorylation of the Ser^78 ^residue of Hsp27 with HER-2/*neu *and lymph node positivity in breast cancer.

## Background

Heat shock proteins (Hsp's) are a large and heterogeneous group of chaperones that include the high-molecular-weight (HMW) Hsp's, such as Hsp70 and Hsp90, and the low-molecular-weight (LMW) Hsp's, including Hsp27 and α-B-crystallin. Hsp synthesis can be induced by both physiological and pathological conditions, such as heat shock, oxidative stress, mitogenic signals, inflammation, infection and neoplastic transformation [1,2]. The HMW Hsp's are involved in protein folding, oligomerization and translocation [3], whereas the LMW Hsp's are related to actin dynamics [4] and to inhibition of apoptosis by interacting with the cytochrome c/Apaf-1/dATP complex in the procaspase-9 pathway or preventing Daxx protein association with Fas and Ask1 [5]. Hsp27 has been found to be overexpressed in breast [6], prostate [7], gastric [8], ovarian [9] and urinary bladder [10] cancers, and its overexpression is associated with aggressive tumor behavior and poor survival rate [11] and adverse resistance to chemotherapy [12]. Hsp27 was also found in the serum of patients with breast cancer and proposed as a possible diagnostic marker for breast cancer [13].

Hsp27 activity is regulated by post-translational modifications such as phosphorylation [3]. Phosphorylation of Hsp27 is catalyzed by MAPKAPK-2 and MAPKAPK-3 [14], protein kinase C (PKC) [15], protein kinase D [16], and cGMP-dependent protein kinase [17]. Endoplasmic reticulum stress induces the phosphorylation of Hsp27 [18] and Stat 3 modulates Hsp27 expression and facilitates phosphorylation at Ser^78 ^[19]. Phosphorylation at its three serine residues (Ser^15^, Ser^78 ^and Ser^82^) induces redistribution of the large oligomers into small tetrameric units [20]. In addition, phosphorylation of Hsp27 results in its translocation from the cytosol to the nucleus and prevents apoptosis [21]. Recently, Shin *et al *[22] found that blocking the phosphorylation of Hsp27 by the specific inhibitor KRIBB3 inhibits tumor cell migration and invasion. In clinical cancer tissues, including renal cell carcinoma [23] and hepatocellular carcinoma [24] and other tissues [25], various phosphorylation patterns of Hsp27 have been found to associate with the aggressiveness of tumor phenotype. For example, attenuated phosphorylation of Hsp27 correlated with tumor progression in hepatocellular carcinoma [24], whereas in renal cell carcinoma, Hsp27 phosphorylation was enhanced, as compared to non-tumor samples [26] and Ser^82 ^was found to be more highly phosphorylated than Ser^15 ^[23]. These apparently paradoxical observations may indicate that phosphorylation of Hsp27 may occur in a tissue- and/or tumor-dependent manner.

In this study, we combined the use of laser capture microscopy (LCM), gel-based proteomics and the phosphosensor dye (Pro-Q Diamond) detection system to identify the differentially phosphorylated phosphoproteins between breast tumors with/without HER-2/*neu *overexpression. The Pro-Q Diamond fluorescence-based system detects phosphoserine-, phosphothreonine- and phosphotyrosine-containing proteins directly in isoelectrofocusing (IEF) gels, SDS-polyacrylamide gels and two-dimensional electrophoresis (2-DE) gels, and has been widely used for phosphoproteomic studies in both cancer cell lines and clinical tumor samples [27-29]. Our comparative phosphoproteomic analyses revealed that Hsp27, one of the identified phosphoproteins, was highly phosphorylated in HER-2/*neu *positive breast tumors. We further investigated the site-specific phosphorylation of Hsp27 at Ser^78^, Ser^82 ^and Ser^15^, with the aim of elucidating the regulatory role of HER-2/*neu*-p38MAPK in Hsp27 phosphorylation and the correlations of their respective pSer profiles with two adverse criteria, HER-2/*neu *and lymph node positivity, associated with tumor progression and poor prognosis. To our knowledge, this is the first report to study the relationship of site-specific phosphorylation of Hsp27 with these two key clinicopathological parameters in breast cancer.

## Results

### Identification of phosphoproteins

**Figure 1:**
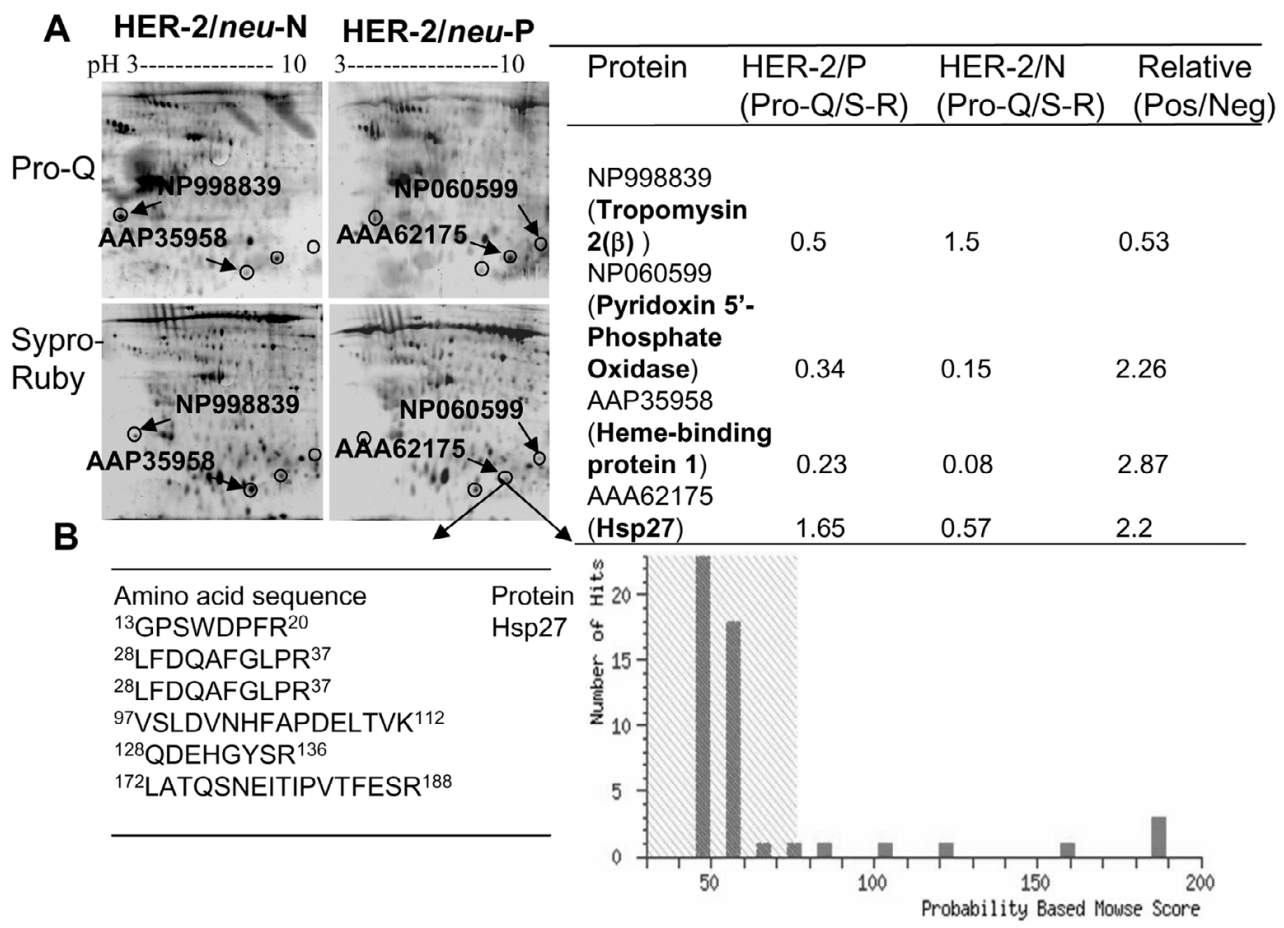
**Identification of phosphoproteins in breast tumors**. (A) Phosphoprotein staining by Pro-Q Diamond and SYPRO-Ruby dyes. Total proteins from both HER-2/*neu *positive and -negative breast tumors were separated by 2-DE gels and stained with fluorescent Pro-Q Diamond for phosphoproteins, followed by SYPRO-Ruby for total proteins. Stained gels were scanned with a Typhoon 9600 fluorescence scanner. Images were captured and the relative phosphorylation levels of differentially phosphorylated spots were analyzed using the ImageMaster 2D Elite software. Proteins were in-gel digested with trypsin, analyzed using 4800 MALDI-TOF/TOF™ analyzer and identified by NCBInr database search. Four phosphoproteins were unambiguously identified (A: in table, right). (B) Peptide sequences and Mowse scores of Hsp27.

**Figure 2:**
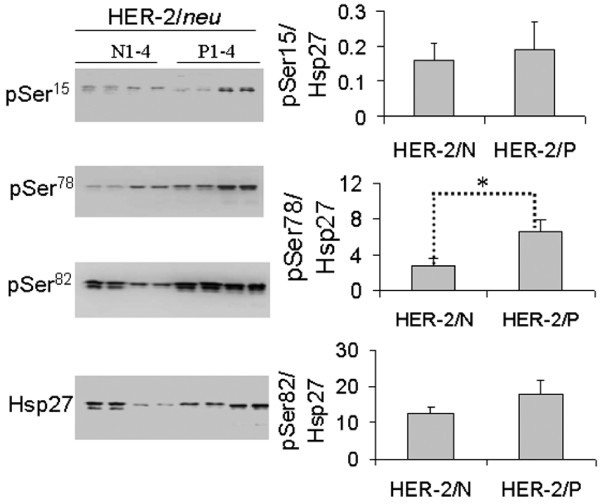
**Western blotting of Hsp27, pSer^15^, pSer^78 ^and pSer^82^**. Twenty μg proteins from each of 4 HER-2/*neu *positive and 4 -negative tumour samples were separated by 10% SDS-PAGE and transferred onto PVDF membranes. After blocking, the membranes were incubated with the respective primary antibodies (anti-Hsp27, anti-pSer^15^, anti-pSer^78 ^and anti-pSer^82^), followed by hybridization to HRP-conjugated secondary antibody. The chemiluminescent signals emitted were captured with the MULTI-GENIUS Bio-Imaging System and signal intensities were analyzed using the GeneTools software (Syngene). The relative phosphorylation levels of pSer^15^, pSer^78 ^and pSer^82 ^presented (histograms, right) are the respective ratios of signal intensity probed with phosphorylation site-specific antibody to signal intensity probed with anti-Hsp27, for each of the three pSer residues. Data with ± SD (standard deviation) are expressed as the average of triplicate experiments. *p < 0.05 (Student *t*-test).

We found significant differences in the phosphoproteomes of HER-2/*neu *positive and – negative tumors. Figure 1A shows an example of 2-DE gels stained by both Pro-Q Diamond and Sypro Ruby. The phosphorylation levels of protein spots were analyzed based on the ratio of spot intensity stained by Pro-Q Diamond over that stained by Sypro Ruby. By using tandem MS/MS peptide sequencing and database search, four differentially phosphorylated proteins were identified as tropomyosin 2(β) (NP998839), pyridoxine 5'-phosphate oxidase (NP060599), Hsp27 (AAA62175) and heme-binding protein 1 (AAP35958). Of these proteins, tropomyosin 2(β) was highly de-phosphorylated, whereas the other three proteins, including Hsp27, were highly phosphorylated in the HER-2/*neu *positive tumors. The peptide sequence and Mowse score of Hsp27 are shown in Figure 1B. As Hsp27 is phosphorylated at three serine residues (Ser^15^, Ser^78 ^and Ser^82^) [3], we further analyzed the levels of site-specific phosphorylation of Hsp27 using the site-specific antibodies on 4 HER-2/*neu *positive and 4 -negative tumors. As shown in Figure 2, residue Ser^78 ^of Hsp27 was highly phosphorylated in HER-2/*neu *positive tumors (*p *< 0.05). There were no significant differences of Hsp27 phosphorylation at Ser^15 ^and Ser^82 ^in the two subtypes of breast tumor cells.

### Ser^78 ^of Hsp27 was highly phosphorylated in HER-2/*neu *positive tumors – tissue lysate array analysis

**Figure 3:**
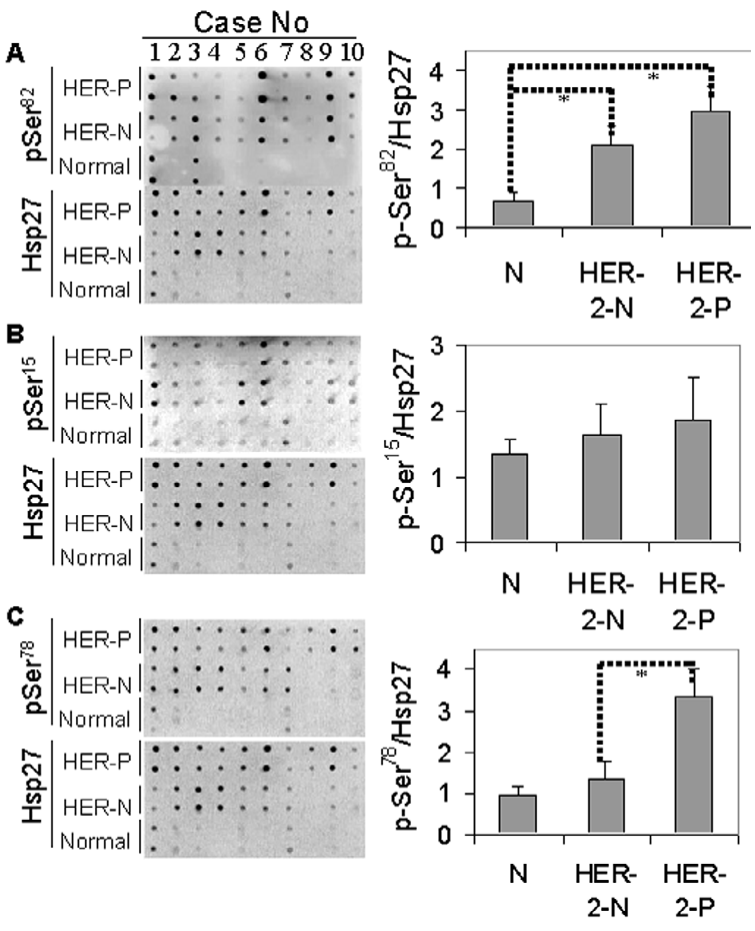
**Tissue lysate array analyses of Hsp27, pSer^15^, pSer^78 ^and pSer^82^**. Equal amounts of total proteins from normal tissues (n = 10), HER-2/*neu *positive (n = 10) and HER-2/*neu *negative (n = 10) breast tumor tissues were manually arrayed on the PVDF membrane in duplicates. Arrays were probed with the respective antibodies and the chemiluminescent signals generated were detected as described in Figure 2. The relative phosphorylation levels (pSer/Hsp27) of Hsp27 at Ser^82 ^(A), Ser^15 ^(B), and Ser^78 ^(C) of each tissue subtype were expressed as the ratio of spot intensity probed with phosphorylation site-specific antibody to the spot intensity probed with anti-Hsp27. The overall average level of pSer/Hsp27 with the standard deviation (± SD), as presented (histograms with error bars) was calculated from 10 cases of each tissue subtype (normal, HER-2/*neu *negative and HER-2/*neu *positive tumor tissues). * Significant differences of three groups (normal, HER-2/*neu *positive and -negative tumors) were calculated using ANOVA. *p < 0.05.

To further confirm the differential phosphorylation of Hsp27 between HER-2/*neu *positive and -negative tumor cells, the site-specific phosphorylations of Hsp27 of HER-2/*neu *positive tumors, -negative tumors and non-tumor tissues were analyzed using a tissue lysate array. As indicated in Figure 3A, the relative level of pSer^82 ^(pSer^82^/Hsp27) was highly increased in both tumor subtypes, as compared to the non-tumor tissues, but there were no significant differences between the HER-2/*neu *positive and -negative tumors. For pSer^15^, no differences were observed between tumor and non-tumor tissues, nor between the two subtypes of breast tumors (Figure 3B). The relative level of pSer^78 ^(pSer^78^/Hsp27) was, however, significantly enhanced in HER-2/*neu *positive tumors (*p *< 0.05), relative to those of HER-2/*neu *negative tumors and non-tumor tissues (Figure 3C). These data imply that HER-2/*neu *signaling plays a key role in Hsp27 phosphorylation at Ser^78^.

### Ser^78 ^phosphorylation was significantly stimulated by heregulin and inhibited by p38 MAPK inhibitor

**Figure 4:**
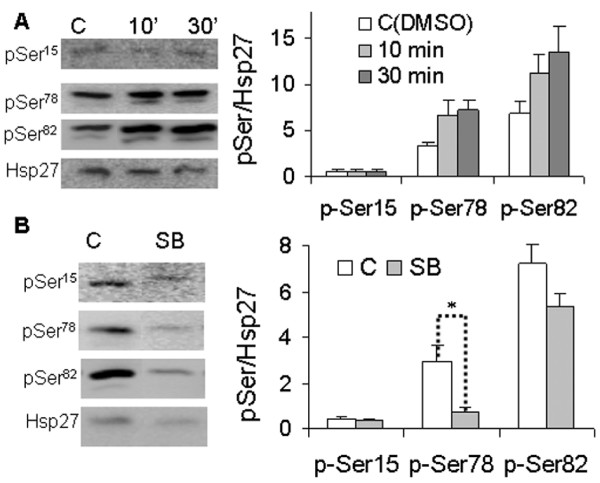
**Effect of heregulin α1 (HRG α1) and p38 MAPK inhibitor (SB 203580) on Hsp27 phosphorylation**. Cultures of cells from the BT474 breast cancer cell line were treated with HRG α1 for 10 and 30 min (A) or SB 203580 for 10 hours (B) and total cell lysates were extracted using M-PER reagent (Pierce). Equal amounts of proteins (20 μg) were separated by SDS-PAGE and transferred onto PVDF membrane. The membranes were blocked for 1 hour, followed by being probed with the respective primary antibodies (anti-Hsp27, anti-pSer^15^, anti-pSer^78 ^and anti-pSer^82^), and HRP-conjugated secondary antibody. The signals were captured and their intensities were detected as described in Figure 2. The phosphorylation levels of pSer^15^, pSer^78 ^and pSer^82 ^were expressed as the ratios of intensity probed with phosphorylation site-specific antibody to the intensity probed with anti-Hsp27. Data with ± SD represents the average of triplicate experiments. C: control; SB: inhibitor SB203580. For the control of HRG-treated cells, untreated cells were cultured for 10 and 30 min and equal amounts of cellular proteins from both time intervals were mixed and used as control. For the control of inhibitor-treated cells, cells were treated with DMSO for 10 hours and cellular proteins were used as the control. *p < 0.05 (student *t*-test)

We further tested the role of HER-2/*neu *signaling in Hsp27 phosphorylation in the BT474 breast cancer cell line by treating the serum-starved cell cultures with HRG α1 for 10 and 30 min. As shown in Figure 4A, HRG α1 significantly stimulates phosphorylation of Ser^78 ^and Ser^82^, but not Ser^15^. As phosphorylation of Hsp27 is the downstream regulator of p38 MAPK [30], we assessed the effect of the p38 MAPK pathway on the Hsp27 phosphorylation profile by inhibiting the p38MAPK pathway with the inhibitor SB203580. Compared to the untreated control, the level of pSer^78 ^was significantly reduced by 70% (*p *< 0.05), whereas that of pSer^82 ^was only inhibited by 30% (Fig. 4B) in the inhibitor-treated cells. The level of pSer^15 ^was not affected by the inhibition of the p38 MAPK pathway. Taken together, these data demonstrate that Ser^78 ^phosphorylation is mainly regulated by the HER-2/*neu*-p38 MAPK pathway and p38 MAPK is the key kinase for Hsp27 phosphorylation at Ser^78^.

### Ser^78 ^phosphorylation of Hsp27 was strongly associated with HER-2/*neu *positivity and lymph node metastasis

**Figure 5:**
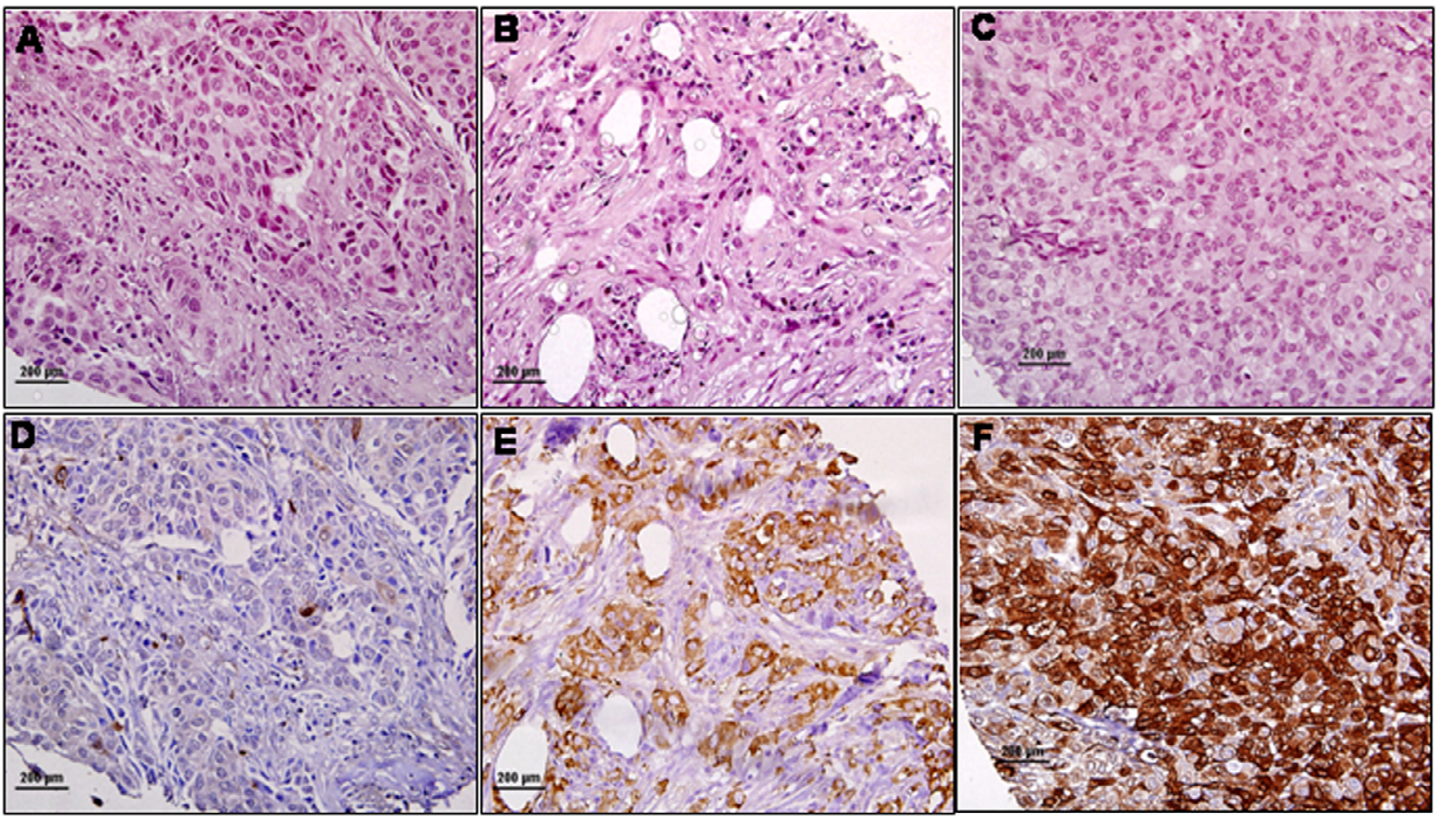
**Representative immunohistochemical staining of pSer^78 ^on breast tumor tissue microarray (TMA)**. TMA sections were deparaffinized in xylene and rehydrated in graded alcohols and processed as described previously [6]. After antigen retrieval and removal of endogenous peroxidases, the sections were incubated for 1 hour with anti-pSer^78 ^antibody and the hybridized complex stained with labeled dextran polymer conjugated with peroxidase and DAB+-substrate chromagen solution, counterstained with Mayer's hematoxylin and mounted for scoring. The extent of staining was scored as negative, weak, moderate and strong, based on the stain intensity. (A-C): Hematoxylin-Eosin (H&E) staining of tumor sample; (D) Negative staining; (E): Moderate staining; (F): Strong staining. Original magnification × 400.

As Hsp27 phosphorylation was involved in tumor cell migration and invasion [22], we then investigated the correlation of pSer^15^, pSer^78 ^and pSer^82 ^with HER-2/*neu *status and lymph node metastasis using immunohistochemical staining on TMA sections comprised of 98 breast tumors. Eighty-nine tumors with available staining information were examined for probable correlations with the specific tumor subgroups, as defined by clinical and pathological variables (e.g., lymph node positivity and HER-2/*neu *status). In all 89 cases, the staining of pSer^78^, pSer^82^, pSer^15 ^and Hsp27 were observed in the cytoplasm. We found that positive anti-pSer^78 ^staining was mostly found in HER-2/*neu *positive tumors (*p *= 0.004) and was strongly correlated with lymph node positivity (*p *= 0.026) (Table 1). Figure 5 shows an example of negative, moderate and strong staining by the anti-pSer^78 ^antibody. We also observed that whilst the extent of Hsp27 staining showed a moderate association with HER-2/*neu *status (*p *= 0.041), no parallel strong correlation with lymph node status (*p *= 0.558) was found. There were also no significant correlations of pSer^15 ^and pSer^82 ^levels with either of these clinicopathological parameters.

**Table 1:** Association of Hsp27 phosphorylation with HER-2/*neu *status and lymph node positivity

	HER-2/*neu *status^*b*^			Lymph node		
Intensity^*a*^	Positive (n = 21)	Negative (n = 68)	*p*	Positive (n = 36)	Negative (n = 53)	*p*

pSer^78^						
N	2	30		8	24	
P	19	38	0.004	28	29	0.026
pSer^82^						
N	8	40		20	28	
P	13	28	0.096	16	25	0.800
pSer^15^						
N	15	53		26	42	
P	6	15	0.539	10	11	0.444
Hsp27						
N	3	26		13	16	
P	18	42	0.041	23	37	0.558

^*a *^staining level was defined as negative for negative/weak staining and positive for moderate and strong staining. P: positive; N: negative. ^*b *^HER-2/*neu *status was described based on FISH: the cutoff value for positive is ≥ 2:1 (HER-2 locus: CEP-17). *p *value was calculated from the chi-square test. Significance was set at *p *< 0.05.

## Discussion

The HER-2/*neu *gene encodes c-ErbB2, a member of the ErbB family of transmembrane tyrosine kinase receptors. Heterodimerization with ErbB3 and ErbB4 and subsequent autophosphorylation of HER-2/*neu *activates the downstream MAPK-Erk1/2 and PI3K-Akt pathways which are central to cell proliferation and survival. The results of our study lend further credence to the view that HER-2/*neu*-mediated phosphorylations of proteins play a key contributory role to poor clinical outcome and resistance to chemo- and hormonal therapies. In this study, we investigated the differential phosphoproteomes by comparing the PRO-Q Diamond stained-phosphoprotein profiles between the LCM-procured HER-2/*neu *positive and -negative tumor cells. Several differentially affected phosphoproteins, including Hsp27, pyridoxine 5'-phosphate oxidase, heme-binding protein 1 and tropomyocin 2(β) were identified. As inhibition of Hsp27 phosphorylation reportedly blocked tumor cell migration and invasion [22], we further extended our work to investigate the association(s) of Hsp27 phosphorylation at three sites (Ser^15^, Ser^78 ^and Ser^82^) with HER-2/*neu *status and lymph node metastasis in breast cancer. To the best of our knowledge, this is the first report showing the strong relationship of pSer^78^, but not pSer^82 ^and pSer^15^, of Hsp27 with HER-2/*neu *status and lymph node positivity in breast cancer.

Hsp27 phosphorylation has been previously reported to be regulated by several pathways, including those of MAPKAPK2/3, PKC/D and Stat3 [14-19]. In this study, to test whether the phosphorylation of Hsp27 at different serine sites was affected by HER-2/*neu *signaling, we treated the BT474 cell line with HRG α1 and with SB203580. Not surprisingly, our data demonstrated that HRGα1 treatment enhanced the phosphorylation of Hsp27 at Ser^78 ^and Ser^82^, though not at Ser^15^. Furthermore, the p38MAPK inhibitor, SB203580, significantly reduced the level of pSer^78 ^(*p *< 0.05), but similar effect on the level of pSer^82 ^was not seen, (Figure 4). These data demonstrated that phosphorylation of the Ser^78 ^residue of Hsp27 was mainly regulated by the HER-2/*neu*-p38 MAPK pathway. Notably, Song *et al*. [19] had found that phosphorylation of the Ser^78 ^residue was mainly induced by Stat3 in MCF-10A and MDA-MB-453 breast cells, further corroborating the role of pSer^78 ^in the aberrant Stat3 signaling-induced cell malignancies. When the MCF-7 cells were treated with the microtubule interfering agent vincristine, phosphorylation of Hsp27 at Ser^78 ^was markedly induced, implying its role in resisting the microtubule dynamic interference by anti-tumoral drugs and enhancing cell survival [31]. As phosphorylation of Hsp27 regulated cell invasion and migration [22,30], our study suggests that the enhanced HER-2/*neu*-p38MAPK-pSer^78^Hsp27 signal could be one of the main regulatory mechanisms in the HER-2/*neu*-driven cell invasion and metastasis.

In studies with resected tumor specimens, it had been found that Hsp27 was highly phosphorylated at Ser^82 ^in renal cell carcinoma [23], whereas in hepatocellular carcinoma, its phosphorylations at three Ser residues were inversely correlated with tumor size, microvascular invasion and tumor stage [24]. In our study, we observed that Ser^78 ^of Hsp27 was highly phosphorylated in HER-2/*neu *positive breast tumors by both Western blot and tumor lysate array analyses (Figures 2 &3). We also showed that immunohistochemical staining intensity strongly correlated to lymph node positivity in the breast tumors tested (*p *= 0.026) (Table 1). These observations indicate that enhanced phosphorylation of Ser^78 ^could be an important effector in driving or facilitating *in vivo *tumor cell invasion and metastasis. However, larger-scale investigations involving more clinical specimens and more extensive clinical evaluations are needed to clarify the exact role of pSer^78 ^of Hsp27 in breast cancer development and progression. From the clinicopathologic point of view, the pSer^78 ^level could serve as a potential biomarker for predicting the extent of malignancy and metastasis in breast cancer.

## Conclusion

Our phosphoproteomics study identified the enhanced phosphorylation of Hsp27 in HER-2/*neu *positive breast tumors. The pSer^78 ^level, in particular, was mainly regulated by the HER-2/*neu*-p38MAPK pathway, and strongly correlated with HER-2/*neu *and lymph node positivity in breast tumors.

## Methods

### Clinical specimens

The HER-2/*neu *status of breast tumors was evaluated by fluorescence *in situ *hybridization (FISH) using the PathVysion kit (Vysis, Downers Grove, IL) and immunohistochemistry (IHC) using the HercepTest Kit (Dako, Glostrup, Denmark), according to the manufacturers' instructions. A cutoff value of ≥ 2:1 signal ratio (HER-2 locus: CEP-17 centromere locus) was defined as HER-2/*neu *gene amplification for FISH, and moderate (+2) to strong (+3) staining of plasma membrane was scored as positive for IHC [32]. For this study, 28 frozen breast tumor tissues (14 HER-2/*neu *positive and 14 HER-2/*neu *negative as determined by FISH and 28 adjacent non-tumor tissues (as controls) were obtained from the Tissue Repository of the Singapore National University Hospital, with informed patient consent. Usage of these tissues complied with the regulations set by our Institutional Review Board (IRB) for research purposes. All the tumors were diagnosed as invasive carcinoma during the diagnostic workup by certified pathologists. Four of the HER-2/*neu*-positive tumors and four of the HER-2/*neu*-negative tumors were used for phosphoproteomic analyses and the rest were used for tissue lysate array analyses.

### Microdissection and protein sample preparation

Tumor cells from 4 HER-2/*neu *positive and 4 HER-2/*neu *negative tumor tissues were dissected using the PixCell II LCM System (Arcturus Engineering, Mountain View, CA), as previously described [6,33]. Cells were immediately lysed in the lysis buffer and the protein concentration of each sample was assayed using the PlusOne 2-D Quantitation Kit (GE Healthcare, San Francisco, CA). To get enough proteins in the samples for 2-DE separations, equal amounts of proteins from each of the 4 HER-2/*neu *positive cases and each of the 4 HER-2/*neu *negative cases were pooled, respectively. The proteins were separated using immobiline IPG DryStrips (180 mm, pH 4–7) and 10% homogeneous SDS-PAGE gels, as described previously [33].

### Phosphoprotein staining and image analysis

Phosphoproteins were stained using the fluorescent Pro-Q Diamond dye, according to the protocol provided by the manufacturer. Briefly, 2-DE gels were fixed overnight in solution containing 45% methanol and 5% acetic acid, followed by washing with deionized water. Gels were incubated in Pro-Q Diamond stain for 2 hours, and destained by washing in 20% acetonitrile in 50 mM sodium acetate (pH 4.0) for 1 hour. After the gels were rehydrated in deionized water for 40 min, the phosphoprotein spots were visualized using the Typhoon 9600 fluorescence scanner (GE Healthcare), with excitation at 532 nm and image capture with the 580 nm long-pass emission filter. Following the phosphoprotein image acquisition, the gels were re-stained for total proteins with SYPRO Ruby and images were acquired again by the same scanner, with excitation at 473 nm and image capture using the 580 nm long-pass emission filter. Computer-generated differential display maps (pseudocolor images) of protein phosphorylation and protein expression patterns were converted into intensity signals and analyzed using the ImageMaster 2D Elite software (GE Healthcare). Spots with at least 1.5-fold changes in the ratio of phosphorylated protein/total protein intensities were excised and digested with sequencing-grade trypsin (Promega, Madison, WI). The trypsinised proteins were analyzed using a 4800 MALDI-TOF/TOF™ analyzer (Applied Biosystems, Foster City, CA) and identified by searching the NCBInr database using the Mascot search program (Matrix Science, London, UK), as previously described [6].

### Cell culture and treatment

The human BT474 breast cancer cell line was obtained from American Type Culture Collection, and maintained in modified Dulbecco's medium (HybriCare) supplemented with 10% fetal bovine serum (FBS) at 37°C in a humidified atmosphere of 5% CO_2 _in an incubator. Prior to HRG α1 (Neomarkers, Fremont, CA) or p38MAPK inhibitor, SB203580 (Calbiochem^®^, Darmstadt, Germany) treatment, the BT474 cells (at ~80% confluence) were serum-starved for 20 hours in medium lacking FBS. For HRG α1 treatment, the starved cells were then exposed to FBS-supplemented medium with 0.3 nM of HRG α1 for 10 min and 30 min. Cells without HRG α1 treatment were cultured for 10 and 30 min, respectively, and equal amounts of total proteins from untreated cells at both time intervals were mixed and used as controls for Western blotting. For the inhibitor treatment, inhibitor SB203580 (5 mg) was dissolved in DMSO to a stock concentration of 40 mM. The starved cells were exposed to FBS-supplemented medium with 40μM of the SB203580 (1:1000 dilution) for 10 hours. The starved cells treated for 10 hrs with equal volume of DMSO were used as control. Total cell lysates were extracted using M-PER reagent (Pierce, Rockford, IL) and their protein concentrations were measured by the Coommassie Plus™ Protein Assay Kit (Pierce).

### Immunoblotting

Cellular proteins (20 μg) were resolved by 10% SDS-PAGE gels and transferred onto PDVF membranes (GE Healthcare). After blocking with 5% bovine serum albumin in Tris-buffered saline containing 0.1% Tween-20, the membranes were incubated overnight at 4°C with the primary antibodies listed in Table 2. The horseradish peroxidase-conjugated goat anti-mouse IgG (1:10,000, Upstate Biotechnology Inc., Lake Placid, NY) or goat anti-rabbit IgG (1:10,000, ZyMED Laboratories Inc., San Francisco, CA) secondary antibody was then applied and the chemiluminescent signals generated using the SuperSignal^® ^West Pico Chemiluminescent Substrate (Pierce) were captured with the MULTI GENIUS Bio Imaging System (Syngene, Frederick, MD) and the signal intensities analyzed using the GeneTools software (Syngene).

**Table 2:** Antibodies used for immunobloting and immunohistochemistry.

Antibodies	Stock conc. (mg/ml)	Dilution	Cat. no.	Sources
Hsp27 (Mouse monoclonal)	0.2	1:1000	Sc-13132	Santa Cruz Biotechnology Inc., Santa Cruz, CA, USA
*p*Ser^78 ^(Mouse monoclonal)	1	1:1000	05–645	Upstate Biotechnology Inc., Lake Placid, NY, USA
*p*-Ser^82 ^(Rabbit polyclonal)	1	1:1000	07–489	Upstate Biotechnology Inc.
*p*-Ser^15 ^(Rabbit polyclonal)	1	1:1000	07–388	Upstate Biotechnology Inc.

Tissue lysate array analysis was carried out as described [33]. Briefly, aliquots of 0.5 μl of each sample were manually spotted onto the PDVF membrane in duplicates. A total of 30 samples – 10 of HER-2/*neu*-positive tumors, 10 of HER-2/*neu*-negative tumors and 10 of non-tumor tissue samples, were arrayed. Treatment of the arrayed membranes and detection of signals with anti-Hsp27 and its phosphorylation site-specific antibodies followed the same procedures as for the immunoblot described above. The relative phosphorylation levels of pSer^15^, pSer^78 ^and pSer^82 ^were individually expressed as pSer/Hsp27: the ratio of spot signal intensity observed when probed with the respective phosphorylation site-specific antibodies to the spot intensity observed when probed with anti-Hsp27 antibody.

### Tissue microarray and immunohistochemistry

Two tissue microarrays (TMAs) containing 97 breast tumors and their corresponding matched, non-tumor controls were constructed previously [34]. To analyze the expression of Hsp27 and its site-specific pSer levels in breast tumors, TMA sections of 4-μm thickness were cut from the TMA block and immunostaining was carried out using the DAKO Envision+ system (Dako, Glostrup, Denmark), as described previously [32]. Briefly, sections were dewaxed in xylene and rehydrated in graded alcohols (100%, 95%, and 75%). Antigen unmasking was done using the DAKO^® ^Target Retrieval Solution in a microwave oven. Endogenous peroxidases were blocked for 1 hour using the supplied Peroxidase block. Sections were incubated for 1 hour with each of the 4 antibodies: anti-Hsp27 (1: 500), anti-pSer^15 ^(1:250), anti-pSer^78 ^(1:250) and anti-pSer^82 ^(1:250), followed by detection with labeled dextran polymer conjugated with peroxidase and DAB^+^-substrate chromagen solution. Three sections were stained with each of the four antibodies. The staining intensities of individual tumor cores on each section were independently scored under a light microscope by a pathologist and the principal researcher (ZD). Cases with discrepant scores were rescored by the same or additional scorers to obtain a consensus score. Staining levels were scored as negative, weak, moderate and strong, based on the staining intensity in the tumor cells. Cases with negative and weak staining intensities were considered as negative; whereas cases with moderate and strong staining intensities were considered as positive. For negative controls, we omitted addition of the primary antibody in the staining protocol.

### Statistical analysis

One-way analysis of variance (ANOVA) was used to compare the significance of differences of Hsp27 phosphorylation levels among the three groups of samples: HER-2/*neu *positive tumors, -negative tumors and non-tumor samples. The correlation of the expression of pSer^15^, pSer^78 ^and pSer^82 ^with the clinicopathologic variables (HER-2/*neu *and lymph node) was analyzed with the chi-square test. Two-sided *p *< 0.05 was considered as of significance.

## Competing interests

The author(s) declare that they have no competing interests.

## Authors' contributions

DZ designed and carried out most of the experiments, performed the statistical analysis, and drafted the manuscript. LLW carried out the experiments involving cell culture and Western blotting. ESK edited the manuscript for publication. All the authors read and approved the final manuscript.
